# Multitarget Strategy for Treatment of Pulmonary Arterial Hypertension: Combination of Mesenchymal Cells with Novel PDE-4 Inhibitor

**DOI:** 10.3390/ph19060907

**Published:** 2026-06-08

**Authors:** Bruno Eduardo Dematté, Juliana Ferreira Vasques, Almir Jordão da Silva-Junior, Lucas Silva Franco, Rodolfo do Couto Maia, Pedro de Sena Murteira Pinheiro, Rosalia Mendez-Otero, Tadeu Lima Montagnoli, Gisele Zapata-Sudo

**Affiliations:** 1 Programa de Pós-Graduação em Cardiologia, Instituto do Coração Edson Saad, Universidade Federal do Rio de Janeiro, Rio de Janeiro 21941-913, RJ, Brazil; brunoeduardodematte@gmail.com; 2 Instituto de Ciências Biomédicas, Universidade Federal do Rio de Janeiro, Rio de Janeiro 21941-902, RJ, Brazilsilvafrancolucas@gmail.com (L.S.F.); rodolfomaia10@yahoo.com.br (R.d.C.M.); pedro_senamp@hotmail.com (P.d.S.M.P.); 3 Instituto de Biofísica Carlos Chagas Filho, Universidade Federal do Rio de Janeiro, Rio de Janeiro 21941-902, RJ, Brazil; jordao.a@biof.ufrj.br (A.J.d.S.-J.); rmotero@biof.ufrj.br (R.M.-O.); 4 Instituto de Química, Universidade Federal do Rio de Janeiro, Rio de Janeiro 21941-909, RJ, Brazil; tlmontagnoli@iq.ufrj.br

**Keywords:** pulmonary arterial hypertension, right ventricle failure, cell therapy, PDE inhibition

## Abstract

**Background**/**Objectives**. Pulmonary arterial hypertension (PAH) is a rare but severe disease which leads to right ventricular (RV) maladaptation, failure and death. Currently approved drugs have limited impact on disease progression. A multitarget strategy consisting of adenosine A_2_B receptor activation and phosphodiesterase-4 (PDE4) inhibition, combined with human mesenchymal stromal cells (hMSCs) therapy, was tested in experimental PAH. The main objective was to determine whether the combination improved pulmonary hemodynamics, vascular remodeling, and RV function, given the limited disease-modifying effects of currently approved vasodilators. **Methods**. Vascular reactivity was assessed in isolated rat pulmonary artery rings exposed to the dual-target compound (LASSBio-1860) alone or in the presence of either ZM-241385 or MRS-1706. PAH was induced in male Wistar rats with monocrotaline (MCT, 60 mg·kg^−1^) and confirmed by a decrease in pulmonary artery acceleration time to ejection time ratio (PAAT/TET). Animals were randomized to receive vehicle, hMSC (single i.v. dose, 1 × 10^5^ cells), LASSBio-1860 (62 mg·kg^−1^·day^−1^*, p.o.,* 14 days), or their combination. Outcomes included PAAT/TET and RV cardiac output (RV-CO) by echocardiography, RV systolic pressure (RVSP) by direct puncture, Fulton index and RV wall thickness, lung histology (perivascular cell counts and wall thickness), and RV protein expression (TGF-β, CaMKII) by Western blot. **Results**. LASSBio-1860 produced endothelium-independent vasorelaxation of rat pulmonary arteries, consistent with A_2_B agonism and PDE4 inhibition. In MCT-induced PAH, combination of LASSBio-1860 and hMSCs resulted in recovery of PAAT/TET and RV-CO, decrease in RVSP, RV hypertrophy, vascular inflammation and remodeling by downregulation of ventricular TGF-β and CaMKII. **Conclusions**. Combination of LASSBio-1860 with hMSC improved RV function, attenuated pulmonary hypertension, RV and vascular remodeling, and reduced inflammatory/proliferative signaling in MCT induced-PAH, supporting a promising multitarget therapeutic strategy for PAH.

## 1. Introduction

Pulmonary arterial hypertension (PAH) is a rare and progressive disorder characterized by pulmonary vascular remodeling and medial hypertrophy. This maladaptive process leads to luminal narrowing of small pulmonary arteries, causing elevated pulmonary vascular resistance and right ventricular (RV) afterload, ultimately leading to right heart failure and death [1]. Precapillary pulmonary vascular remodeling is a hallmark of PAH and contributes to the increase in mean pulmonary arterial pressure (mPAP > 20 mmHg at rest) despite normal pulmonary capillary wedge pressure [2]. Moreover, current pharmacotherapy focuses mainly on hemodynamic improvement through pulmonary vasodilation while insufficiently addressing the mechanisms involved in disease progression. Consequently, the prognosis remains poor, with mortality rates still approaching 50% within seven years [3].

Five drug classes are approved for clinical use in PAH—calcium channel blockers, phosphodiesterase-5 inhibitors, endothelin receptor antagonists, prostacyclin receptor agonists, and guanylate cyclase stimulators—but monotherapy is frequently limited by modest efficacy and significant adverse effects [4]. Endothelin receptor antagonists are associated with hepatotoxicity and peripheral edema [5]; prostacyclin analogs often cause systemic hypotension [6]; and phosphodiesterase-5 inhibitors can cause headaches and visual disturbances [7]. These adverse events affect treatment adherence and quality of life of patients [8].

Therapies approved for PAH have been largely centered on pulmonary vasodilation, aiming to improve symptoms and hemodynamic parameters. However, increasing experimental and clinical evidence indicates that the key pathophysiological processes involved in PAH progression—such as endothelial-to-mesenchymal transition (EndMT) [9], inflammation and fibrosis [10,11]—are not fully addressed by vasodilator-based strategies alone. Therefore, a multitarget drug strategy represents an alternative to overcome such limitations by modifying both hemodynamic and histologic alterations observed in PAH. In this context, a new compound, named LASSBio-1860, designed as dual phosphodiesterase-4 (PDE4) inhibitor and adenosine A_2_ receptor agonist [12,13], was proposed as a potential therapy. LASSBio-1860 represents a potential dual-mechanism therapy aimed at modifying disease progression through simultaneous modulation of proliferative and inflammatory pathways.

To further enhance therapeutic efficacy, the combination of LASSBio-1860 with human mesenchymal stem cells (hMSCs) may provide synergistic benefits by integrating pharmacological and cell-based approaches to attenuate vascular remodeling while promoting cardioprotection [14]. The present work investigated the effects of LASSBio-1860 in combination with hMSC in a monocrotaline (MCT)-induced rat model of PAH, including hemodynamic, morphometric, and molecular parameters relevant to disease progression.

## 2. Results

### 2.1. Multitarget Profile of LASSBio-1860

**Figure 1:**
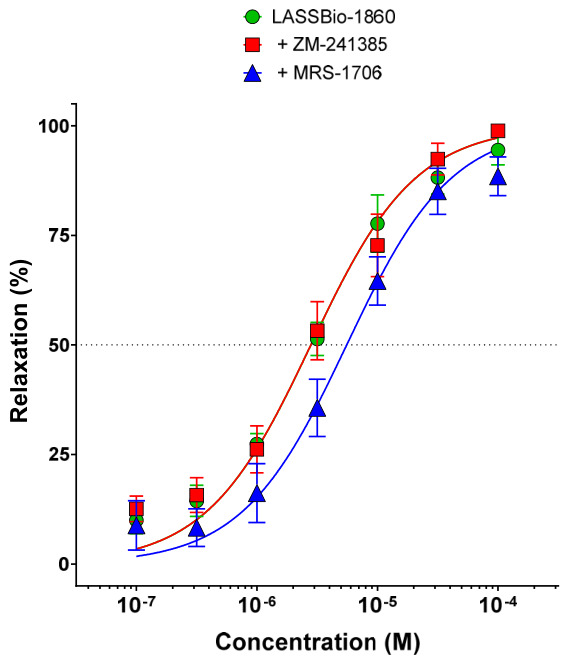
Relaxation of endothelium-denuded pulmonary artery rings induced by LASSBio-1860. Tissues were incubated with vehicle (*n* = 5), ZM-241385 (300 nM; *n* = 7), or MRS-1706 (100 nM; *n* = 6). LASSBio-1860 induced concentration-dependent relaxation (EC_50_ 2.80 ± 0.29 μM), which remained unchanged in the presence of A_2A_ antagonist ZM-241385 (EC_50_ 2.80 ± 0.35 μM). However, A_2B_ antagonist MRS-1706 increased the EC_50_ to 5.55 ± 0.78 μM (*p* < 0.05). Data are presented as mean ± SEM.

The proposed mechanism of action of LASSBio-1860 was initially assessed in isolated rat pulmonary artery rings. LASSBio-1860-induced relaxation of pulmonary arteries was not altered by mechanical removal of the endothelium, suggesting that its effects are endothelium-independent. As shown in Figure 1, LASSBio-1860 induced a concentration-dependent vasorelaxation (EC_50_ 2.80 ± 0.29 μM), which remained unchanged in the presence of 300 nM ZM-241385 (EC_50_ 2.80 ± 0.35 μM), suggesting minimal involvement of A_2A_ receptors. In contrast, exposure to 100 nM MRS-1706, an A_2B_ receptor antagonist, produced a rightward shift in the concentration–response curve, resulting in a significant increase in EC_50_ (5.55 ± 0.78 μM, *p* < 0.05). Therefore, these findings suggest that the vasodilatory effects of LASSBio-1860 in pulmonary arteries are predominantly mediated by A_2B_ rather than A_2A_ adenosine receptor activation.

**Figure 2:**
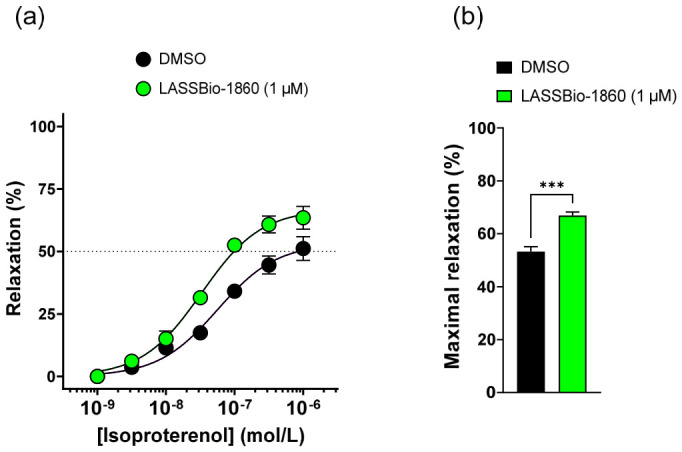
Effect of LASSBio-1860 on isoproterenol-induced vasorelaxation in pulmonary artery rings. (**a**) Rings were incubated with vehicle (*n* = 5) or LASSBio-1860 (1 μM; *n* = 5) and exposed to cumulative concentrations of β-adrenergic agonist. Isoproterenol promoted concentration-dependent relaxation with an EC_50_ of 56.17 ± 7.46 nM (vehicle) and 33.17 ± 2.79 nM (LASSBio-1860), indicating enhanced β-adrenergic vasorelaxation in the presence of LASSBio-1860. (**b**) Maximal relaxation (%) induced by isoproterenol in the presence of vehicle or LASSBio-1860. Bars represent mean ± SEM (*n* = 5/group); *** *p* < 0.001 vs. vehicle (unpaired two-tailed *t*-test). Data are presented as mean ± SEM.

To further assess the role of PDE inhibition-mediated 3′,5′-cyclic adenosine monophosphate (cAMP)/protein kinase A (PKA) activation in the observed effects, pulmonary artery rings were preincubated with LASSBio-1860 or vehicle and their vasodilation to isoproterenol was compared (Figure 2a). LASSBio-1860 produced a leftward shift in the isoproterenol concentration–relaxation curve, with a decrease in EC_50_ from 56.17 ± 7.46 to 33.17 ± 2.79 nM. In addition, maximal relaxation induced by isoproterenol was increased in the presence of LASSBio-1860 (Figure 2b).

β-adrenergic agonists such as isoproterenol relax the pulmonary arteries activating adenylyl cyclase (AC), thereby increasing intracellular cAMP levels, which may be further enhanced by PDE4 inhibition, leading to enhanced pulmonary artery relaxation. Thus, elevation of cAMP, either by stimulating its synthesis or by reducing its degradation, represents an efficient strategy to enhance pulmonary vasodilation.

### 2.2. Combination Therapy of LASSBio-1860 and hMSCs

**Figure 3:**
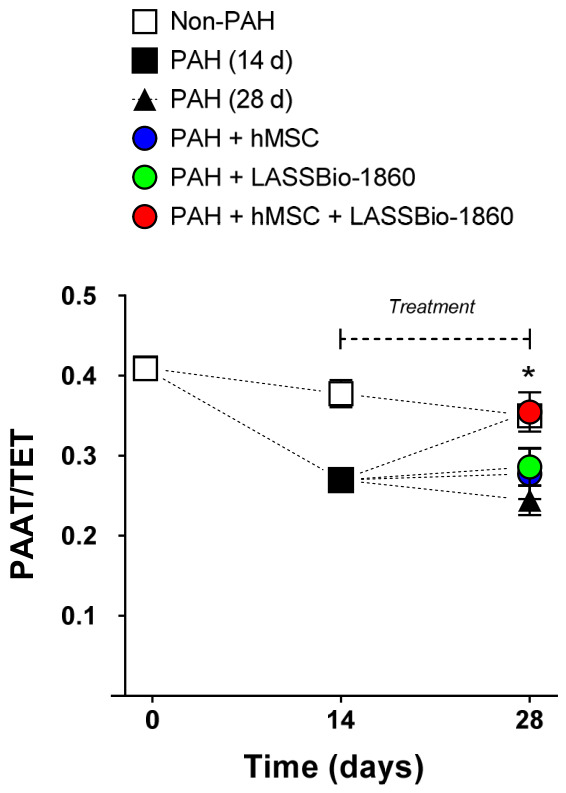
Echocardiographic assessment of PAAT/TET across disease progression and treatment. The pulmonary artery acceleration time/ejection time ratio (PAAT/TET) was measured by pulsed-wave Doppler at baseline (day 0), after establishment of PAH (day 14), and following a 14-day treatment period (day 28). From day 14 to 28, animals received: vehicle (PAH), hMSCs (single administration, 1 × 10^5^ cells), LASSBio-1860 (62 mg·kg^−1^·day^−1^, p.o.), or their combination. Mean ± SEM values were: baseline, 0.41 ± 0.06; PAH (14 d), 0.27 ± 0.01; PAH (28 d), 0.26 ± 0.01; PAH + hMSCs, 0.27 ± 0.01; PAH + LASSBio-1860, 0.28 ± 0.01; PAH + hMSCs + LASSBio-1860, 0.35 ± 0.02. PAH reduced PAAT/TET from baseline to day 14. Combination therapy increased PAAT/TET compared with untreated PAH (* *p* < 0.05 vs. PAH, 28 d; *n* = 5–7).

After 14 days of PAH induction, PAAT/TET declined from 0.41 ± 0.06 to 0.27 ± 0.01, as shown in Figure 3. At the end of the protocol, isolated therapies produced only modest, non-significant increases in PAAT/TET, reaching 0.27 ± 0.01 and 0.28 ± 0.01 in the hMSCs and LASSBio-1860 groups, respectively. The combination of hMSCs + LASSBio-1860 elevated PAAT/TET to 0.35 ± 0.02, significantly higher than the 0.26 ± 0.01 observed in untreated PAH rats (*p* < 0.05).

**Figure 4:**
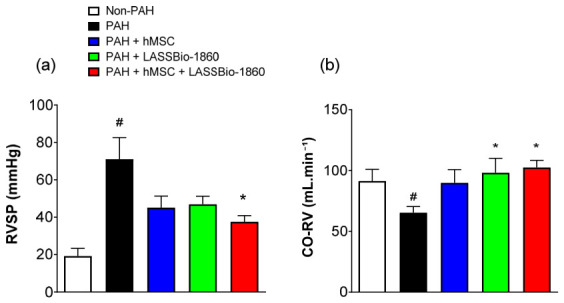
Effects of LASSBio-1860 and hMSCs on right ventricular hemodynamics in PAH. (**a**) Right ventricular systolic pressure (RVSP, in mmHg) measured by right ventricular puncture (mean ± SEM): Non-PAH, 19.21 ± 4.15; PAH, 71.03 ± 11.53; PAH + hMSCs, 45.09 ± 6.22; PAH + LASSBio-1860, 46.94 ± 4.30; PAH + hMSCs + LASSBio-1860, 37.58 ± 3.27. (**b**) Right ventricular cardiac output (RV-CO, mL·min^−1^) was assessed by echocardiography at the conclusion of the protocol: Non-PAH, 91.27 ± 9.67; PAH, 65.22 ± 5.19; PAH + hMSCs, 89.82 ± 10.94; PAH + LASSBio-1860, 98.11 ± 11.89; PAH + hMSCs + LASSBio-1860, 102.40 ± 5.98 (# *p* < 0.05 vs. Non-PAH; * *p* < 0.05 vs. PAH).

As presented in Figure 4a, PAH induction resulted in an increase in right ventricular systolic pressure (RVSP) from 19.21 ± 4.15 mmHg (control group) to 71.03 ± 11.53 mmHg. Either hMSCs or LASSBio-1860 reduced RVSP to 45.09 ± 6.22 mmHg and 46.94 ± 4.30 mmHg. Combined treatment with hMSCs and LASSBio-1860 significantly reduced RVSP to 37.58 ± 3.27 mmHg. Figure 4b shows right ventricular cardiac output (RV-CO), assessed by echocardiography. The PAH group exhibited a marked decrease in RV-CO of 65.2 ± 5.2 mL·min^−1^ compared to the Non-PAH group, 91.3 ± 9.7 mL·min^−1^, indicating disease-induced cardiac dysfunction. Recovery of RV-CO to 102.4 ± 5.9 mL·min^−1^ was observed after treatment with the combination of hMSCs and LASSBio-1860. Administration of LASSBio-1860 or hMSCs alone restored RV-CO to levels close to the control group, reaching 98.1 ± 11.9 and 89.8 ± 10.9 mL·min^−1^, respectively.

**Figure 5:**
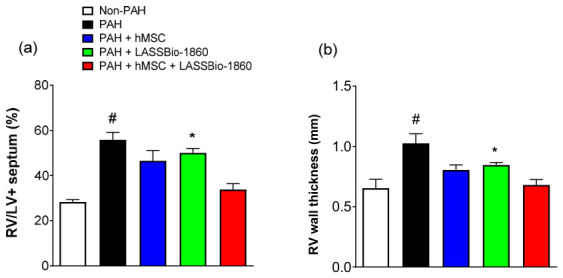
Right ventricular remodeling in monocrotaline-induced PAH and after treatment. (**a**) Fulton index (RV/LV + S, %). (**b**) RV anterior wall thickness in diastole (mm) was assessed echocardiographically. Bars represent mean± SEM. One-way ANOVA with Tukey’s *post hoc* test. # *p* < 0.05 vs. Non-PAH; * *p* < 0.05 vs. PAH (*n* = 5–7).

In addition to RV functional evaluation, structural parameters were also investigated. MCT produced marked RV remodeling and hypertrophy relative to the Non-PAH group, increasing the Fulton index from 28.3 ± 1.0% to 55.8 ± 3.3% and RV anterior wall thickness from 0.65 ± 0.07 to 1.02 ± 0.07 mm (Figure 5). In contrast, the combined treatment (hMSCs + LASSBio-1860) significantly reduced both markers of RV remodeling compared with PAH (Fulton 33.8 ± 2.6%; RV thickness 0.68 ± 0.04 mm; *p* < 0.05). These data indicate that combined hMSCs and LASSBio-1860 therapy was more effective in reducing RV remodeling than either treatment alone.

**Figure 6:**
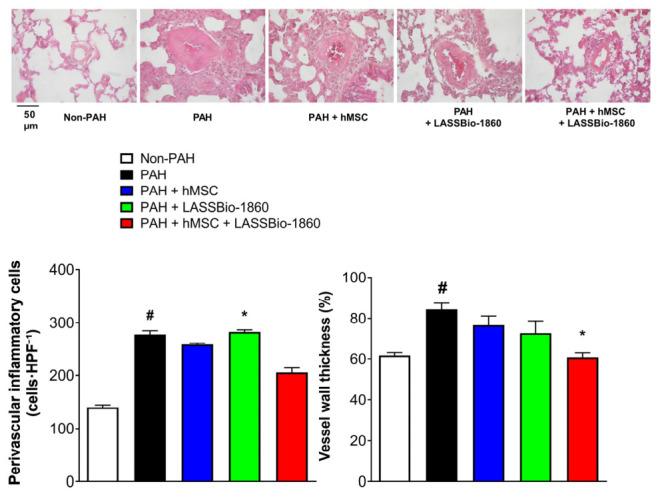
Attenuation of PAH-induced perivascular inflammation and vascular remodeling by combined therapy. H&E-stained lung sections illustrating perivascular inflammatory infiltrates and medial thickening in PAH animals, with attenuation after combined therapy. Perivascular inflammatory cells (cells·HPF^−1^) counted at 400× within one vessel diameter of the external elastic lamina. PAH increased perivascular cell counts versus Non-PAH (# *p* < 0.05); combined treatment significantly reduced infiltration compared with PAH (* *p* < 0.05). Bottom, right: Vessel wall thickness (% of vessel diameter). PAH increased wall thickness relative to Non-PAH (# *p* < 0.05). The combined therapy (hMSCs + LASSBio-1860) markedly decreased wall thickness versus PAH (* *p* < 0.05). Bars show mean ± SEM; groups were compared by one-way ANOVA followed by Tukey’s *post hoc* multiple comparisons.

Histopathological analysis (Figure 6) corroborated the anti-remodeling effect of the combined regimen. Hematoxylin–eosin-stained lung sections from PAH animals exhibited a dense perivascular inflammatory infiltrate and medial hypertrophy relative to Non-PAH. In contrast, the combined therapy (hMSCs + LASSBio-1860) significantly attenuated inflammatory infiltration and reduced vascular wall thickness compared with untreated PAH. These findings indicate that the combination strategy mitigates both the inflammatory and structural components of pulmonary vascular remodeling in this model.

**Figure 7:**
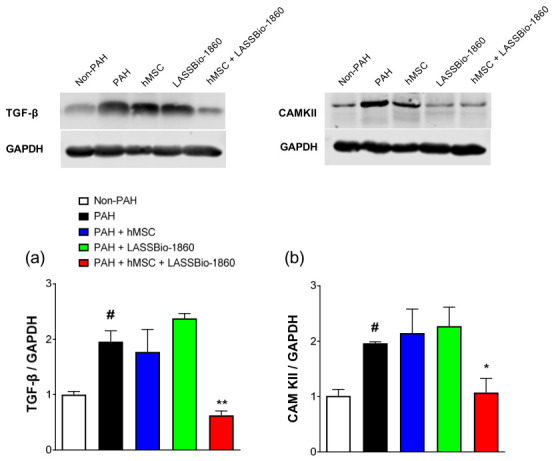
Combined treatment with LASSBio-1860 and hMSCs reduces profibrotic and proliferative signaling in cardiac tissue. Western blot quantification in RV myocardium (normalized to GAPDH) shows that PAH increases TGF-β (**a**) and CaMKII (**b**) expression compared with Non-PAH controls (# *p* < 0.05). Neither hMSCs alone nor LASSBio-1860 alone attenuated these elevations. In contrast, the combined treatment (hMSCs + LASSBio-1860) markedly reduced both TGF-β and CaMKII to levels significantly lower than PAH (* *p* < 0.05; ** *p* < 0.01 vs. PAH). Bars represent mean ± SEM (*n* = 3/group).

In cardiac tissue, Western blot normalized to GAPDH demonstrated that PAH markedly increased the expression of both transforming growth factor (TGF)-β and Ca^2+^/calmodulin-dependent protein kinase type II (CaMKII) compared with the Non-PAH control. Neither hMSCs monotherapy nor LASSBio-1860 treatment alone reduced these elevations. In contrast, the combined treatment (hMSCs + LASSBio-1860) produced a robust reduction in TGF-β and CaMKII density, significantly lower than in PAH (Figure 7). TGF-β is associated with cardiac fibrosis and fibroblast activation under RV pressure overload and PAH, while CaMKII is a key mediator of pathological Ca^2+^ signaling in myocardium, contributing to hypertrophy, inflammation, and adverse remodeling. These data indicate that combined hMSCs and LASSBio-1860 treatment more effectively attenuates profibrotic (TGF-β) and pro-remodeling (CaMKII) signaling than either intervention alone.

## 3. Discussion

The pathogenesis of PAH is complex and multifactorial, involving endothelial cell dysfunction [15], proliferation of pulmonary arterial smooth muscle cells (PASMCs) [16], adventitial fibroblast activation, and inflammation, which collectively contribute to vascular remodeling, medial hypertrophy, and vessel obstruction [17]. 

In the pulmonary vasculature, activation of G protein-coupled adenosine receptors A_2A_ and A_2B_ promotes vasodilation through stimulation of adenylyl cyclase, and increased intracellular cAMP [18]. In particular, adenosine receptor A_2B_ signaling has been implicated in the reduction of smooth muscle cell proliferation and attenuation of vascular remodeling [19,20]. Moreover, PDE4 regulates intracellular cAMP hydrolysis, and its inhibition increases cAMP/PKA signaling, resulting in pulmonary vasodilation and anti-proliferative effects in PASMCs [21]. In addition, PDE4 has been implicated in the modulation of EndMT, a critical contributor to pulmonary vascular remodeling and disease progression, reinforcing its relevance as a therapeutic target in PAH [22]. PDE4 is also the dominant isoenzyme in inflammatory cells, and its inhibition could attenuate transforming growth factor beta (TGF-β) signaling via cAMP/PKA activation in human lung fibroblasts, indicating broader antifibrotic and anti-inflammatory potential [23,24].

Emerging strategies have been proposed using hMSCs due to their efficacy in modulating pulmonary vascular paracrine mediators [25]. Preclinical studies using MSCs and their extracellular vesicles demonstrated anti-inflammatory and antiproliferative effects on the pulmonary vasculature and a protective effect on RV [26]. Hence, MSCs represent a potential approach to address the multiple processes involved in PAH pathogenesis [27]. Preclinical studies demonstrate that MSCs and their extracellular vesicles (EV) [28] promote beneficial changes in pathophysiology in PAH [29], providing benefits beyond vasodilation [26]. MSC-based products prevent or reverse pulmonary vascular remodeling, improve hemodynamics and RV function, and reduce perivascular inflammation [30,31].

Our data suggest a new, multitarget disease-modifying approach for PAH. LASSBio-1860 induced endothelium-independent relaxation of isolated pulmonary arteries through a dual mechanism involving activation of A_2B_ adenosine receptor and inhibition of PDE4. Moreover, its combination with hMSCs improved pulmonary and cardiac outcomes in monocrotaline-induced PAH as demonstrated by reduced RVSP and RV hypertrophy, restored RV-CO, and attenuated perivascular tissue inflammation and pulmonary vascular thickness. These effects were accompanied by reduced TGF-β and CaMKII content in RV myocardium, indicating suppression of important inflammatory, profibrotic and proliferative markers in this animal model of PAH [32,33,34,35]. LASSBio-1860 offers a new dual cAMP-elevating mechanism, through A_2_B receptor activation and PDE4 inhibition, thereby potentiating the paracrine immunomodulatory and anti-proliferative actions of hMSCs to improve pulmonary vascular remodeling and RV maladaptation. The combined regimen achieved greater improvements in hemodynamic and structural markers than either monotherapy.

Our findings are in accordance with preclinical studies, in which the activation of A_2_B adenosine receptor reduces PASMC growth [19]. Activation of A_2_B receptor in the pulmonary artery leads to the amplification of cAMP/PKA signaling, which confers anti-mitogenic properties [18]. Interestingly, some studies report an increased expression of A_2_B adenosine receptor in lungs of PAH patients [36,37], while preclinical studies have suggested a context-dependent activation in tissue macrophages which promotes lung fibrosis and PH [38]. However, PDE4 inhibition also increases cAMP/PKA-dependent vascular muscle relaxation [39], regulates baseline sarcoplasmic reticulum Ca^2+^ release and cardiac contractility [40], counterbalances proliferative/inflammatory mechanisms in PASMCs [41], downregulates the fibroblast-to-myofibroblast transition and proliferative activity in lung fibroblasts [35], and, particularly PDE4B, regulates EndMT [22] and vascular inflammation in the lungs.

The reprogramming of immune and vascular pathways promotes suppression of NF-κB through PDE inhibition and PKA phosphorylation, thereby limiting smooth-muscle proliferation [42]. These effects provide a biologic rationale for pairing hMSC-mediated paracrine immunomodulation altogether with pharmacological agents to interfere with remodeling. Positive results in clinical studies with umbilical cord MSC-derived products, in severe pediatric PAH, reinforce the translational development [43]. The combined action of LASSBio-1860 and the anti-inflammatory properties of cellular therapy [44] represent a novel approach to mitigate cardiac and pulmonary vascular remodeling, in addition to causing vascular relaxation.

The combined therapy used targets both the vascular (remodeling and inflammation) and the cardiac (RV dysfunction and remodeling) components of PAH. LASSBio-1860 could promote cAMP elevation by enhancing its synthesis and inhibiting its degradation, thus inducing pulmonary vasodilation. In addition, elevated cAMP could counteract proliferative and inflammatory pathways by regulating perivascular inflammation and reducing vascular hyperplasia and extracellular matrix deposition, which play an important role in PAH progression. Therefore, the combination of increased intracellular cAMP and paracrine immunomodulation by hMSCs emerges as a promising multitarget approach to mitigate structural and inflammatory alterations involved in PAH progression.

Although the MCT-induced PAH has proved to represent a favorable model to investigate pulmonary vascular remodeling, inflammation and RV function, it does not fully characterize the biological heterogeneity of the human disease. Future studies should evaluate alternative therapeutic regimen, long-term efficacy, and potential adverse effects. Finally, the combination of the increase in cAMP and hMSC-based therapy may contribute to disease modification in PAH. These findings indicate that the elevation of cAMP, through both A_2_B receptors and PDE4 inhibition, in combination with the paracrine immunomodulatory hMSCs actions [45], yields beneficial effects on both pulmonary vascular and RV remodeling, and this integrated approach represents a promising disease-modifying therapeutic alternative for PAH.

## 4. Materials and Methods

### 4.1. Drugs and Reagents

**Figure 8:**
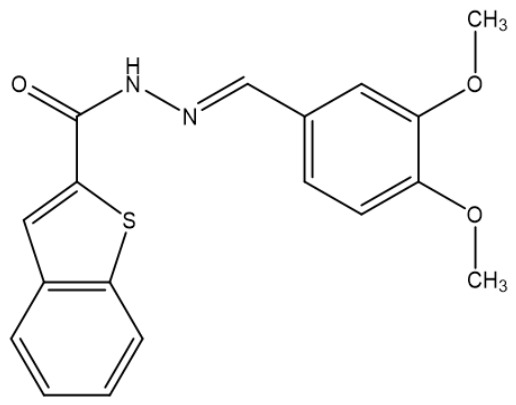
Chemical structure of LASSBio-1860, an *N*-acylhydrazone derivative reported to inhibit PDE4 catalytic activity by 70% and to exhibit 10 µM affinity at adenosine A2 receptors, supporting its dual-mechanism for PAH therapy [13].

(*E*)-*N′*-3,4-dimethoxybenzylidenebenzo[b]thiophene-2-carbohydrazide, named LASSBio-1860 (Figure 8), was synthetized at Laboratório de Avaliação e Síntese de Substâncias Bioativas, Universidade Federal do Rio de Janeiro, Brazil. Ketamine and isoflurane were provided by Cristália Produtos Químicos e Farmacêuticos Ltd.a (Itapira, SP, Brazil). Isoproterenol and monocrotaline were purchased from Sigma-Aldrich (St. Louis, MO, USA).

### 4.2. Animals and Experimental Design

All experimental protocols were approved by the Committee on Ethics in the Use of Animals at Universidade Federal do Rio de Janeiro (license number 018/23). Male Wistar rats (200–250 g) were kept under a light/dark cycle of 12 h at 24 °C, with free access to water and pellet-type feed.

### 4.3. Vascular Reactivity of Pulmonary Artery Rings

Pulmonary arteries were carefully dissected from euthanized Wistar rats and placed in a physiological solution (composition in mM: NaCl 123, KCl 4.7, CaCl_2_ 1.2, MgSO_4_ 1.2, KH_2_PO_4_ 1.2, NaHCO_3_ 15.5 and glucose 11.1), pH 7.4, maintained at 37 °C, and oxygenated with 95% O_2_/5% CO_2_. Vessels were cleaned of connective tissue and rings (2–3 mm in length) were obtained with or without intact endothelium, confirmed by lack of acetylcholine-induced relaxation (10 µM) in precontracted aorta (with phenylephrine, 10 µM). Rings were positioned in organ baths for isometric tension recording using force transducers connected to Lab Chart software (Version 7.0, ADInstruments, Inc., Sydney, Australia). After 60 min under a resting tension of 1.5 g, rings were exposed to cumulative concentrations of LASSBio-1860 (10^−7^ to 10^−4^ M) to obtain concentration–response curves. Intact endothelium was confirmed when rings relaxed >80% in response to acetylcholine but the absence was considered when response was <10%. Data were expressed as percentage of vascular relaxation relative to maximal contraction induced by phenylephrine. Fifty percent of the concentration to induce vascular relaxation was determined for LASSBio-1860 (EC_50_) in the absence and presence of previous exposure (30 min) to pharmacological antagonists of adenosine A_2_A and A_2_B receptors, ZM-241385 (3 µM) and MRS-1706 (1 µM), respectively. To investigate the involvement of PDE and cAMP formation pathways, rings were preincubated for 30 min with LASSBio-1860 (1 µM) followed by exposure to cumulative concentration of isoproterenol (10^−9^ to 10^−6^ M).

### 4.4. PAH Induction

PAH was induced in male Wistar rats (200–250 g) by a single intraperitoneal injection of monocrotaline (MCT, 60 mg/kg). Two weeks after PAH induction, the increase in mPAP was confirmed using transthoracic echocardiography, based on the increase in pulmonary artery acceleration time and total ejection time ratio (PAAT/TET). After confirmation, animals were randomly divided into five experimental groups: (1) control, (2) PAH + vehicle, (3) PAH + LASSBio-1860 (62 mg/kg/day p.o.), (4) PAH + hMSC (10^5^ cells) and (5) PAH + LASSBio-1860 + hMSC. A single dose of 10^5^ hMSC was intravenously administered into the caudal vein followed by 14 days of oral treatment with LASSBio-1860. Vehicle groups received the same volume (300 μL) of DNase I in PBS (0.6 U/mL). Umbilical cord-derived hMSCs were isolated and immunophenotyped following Alencar et al. [44].

### 4.5. Transthoracic Echocardiography

Transthoracic echocardiography was performed under anesthesia with isoflurane 1.5% using an ultrasound system (Philips CX50 Healthcare, Andover, MA, USA) coupled to a 12–4 MHz probe. Animals were placed in the supine position on a heated platform, and heart rate was monitored throughout the procedure. Parasternal long-axis and short-axis images were obtained, along with pulmonary artery doppler measurements to determine PAAT/TET and confirm PAH. After 14 days of treatment, echocardiography was repeated to obtain the following hemodynamic parameters: PAAT/TET, cardiac output and RV anterior wall thickness. Parameters of left-ventricular structure and function were obtained to exclude left-sided heart failure and post-capillary pulmonary hypertension.

### 4.6. Cardiac Catheterism

Animals were anesthetized with ketamine (80 mg/kg) and xylazine (15 mg/kg), and using the open-chest approach, RV pressure was directly measured by ventricular puncture through a needle connected to a pressure transducer interfaced with Lab Chart software (Version 7.0, ADInstruments, Inc.). RVSP measurement using direct RV puncture confirmed the RV overload at the endpoint of PAH evaluation [46,47,48]. Although this approach did not provide comprehensive information about hemodynamic status, it was important to further assess RV remodeling demonstrated by echocardiography analysis.

### 4.7. Morphometric and Histological Analysis

At the end of experimental protocol, animals were euthanized, and tissues were weighed and prepared for histological and protein expression analyses. The ratio of RV and left ventricle plus septum weight (RV/LV + S) was calculated to assess RV hypertrophy (Fulton index). Cardiac tissues were stored at −80 °C for subsequent protein expression analyses, while pulmonary tissue was fixed in a 10% formaldehyde solution and embedded in paraffin for histological evaluation. Paraffin-embedded samples were sectioned at 5 µm using a microtome (Lupe, MRP-03, São Carlos, SP, Brazil) and inflammatory infiltrates were assessed on hematoxylin–eosin-stained sections. Cells were counted at 400× within one vessel diameter of the external elastic lamina (cells·HPF^−1^). Interstitial cells were examined in ten fields per slide with a digital camera (Canon, Melville, NY, USA) coupled to a light microscope at 400× magnification (Axiostar plus, Zeiss, Jena, Germany). Using the same histological staining and method, the vessel wall thickness was obtained by calculating the percentage of vessel diameter over the total external area.

### 4.8. Membrane Preparation and Western Blot

Heart samples were rapidly excised and frozen in liquid nitrogen. For protein extraction, tissues were kept on ice and mechanically homogenized with lysis buffer (12.5% sucrose, 20 mM Tris-HCl, pH 7.4, 1 mM EDTA) supplemented with protease inhibitors (1 mM phenylmethylsulfonyl fluoride, 1 mM benzamidine, 1 mM dithiothreitol, 1 µg/mL pepstatin A, 1 µg/mL chymostatin, 1 µg/mL aprotinin, 1 µg/mL leupeptin, and 1 µg/mL antipain). Homogenates were clarified by centrifugation (5 min, 1000× *g*, 4 °C); supernatants were collected and stored at −80 °C. Protein concentration was determined by the Coomassie (Bradford) method using bovine serum albumin standards. Aliquots were mixed 1:1 with 2× Laemmli sample buffer containing 5% β-mercaptoethanol, heated for 5 min at 95 °C, and equal amounts of total protein (50 µg per lane) were resolved on 10% SDS–polyacrylamide gels. Proteins were transferred to nitrocellulose membranes using a semi-dry apparatus (Bio-Rad, Hercules, CA, USA). Transfer quality and equal loading were verified by Ponceau S staining. Membranes were blocked for 1 h at room temperature in PBS containing 5% non-fat dry milk and 0.1% Tween-20 (PBS-T), then incubated overnight at 4 °C with primary antibodies diluted in PBS-T: TGF-β (1:1000), CAMKII (1:1000), and anti-GAPDH (1:1000). After three washes in PBS-T (5 min each), membranes were incubated for 1 h at room temperature with HRP-conjugated secondary antibodies (1:10,000 in PBS-T), washed again, and developed by enhanced chemiluminescence. Chemiluminescent detection of specific bands was captured on an ImageQuant LAS-4000 system (GE Healthcare, Chicago, IL, USA).

### 4.9. Statistical Analysis

Data are expressed as the mean ± SEM. The one-way ANOVA followed by Tukey’s post hoc test was used for comparison (GraphPad Prism, version 6, San Diego, CA, USA). Differences between the experimental groups were considered statistically significant at *p* < 0.05.

## 5. Conclusions

In conclusion, the combination of LASSBio-1860 and hMSCs improved RV function, attenuated pulmonary and RV remodeling, and reduced inflammatory and proliferative signaling in the monocrotaline-induced PAH model. These findings provide a novel preclinical pharmacological–cell-based therapeutic strategy for PAH.

## Data Availability

The original contributions presented in this study are included in the article. Further inquiries can be directed to the corresponding author.
